# A preliminary study of implicit statistical learning in non-clinical young adults exposed to early adversity

**DOI:** 10.3389/fpsyt.2026.1904450

**Published:** 2026-08-13

**Authors:** Paweł Krukow, Natalia Kopiś-Posiej, Víctor Rodríguez-González, Víctor Gutiérrez-de Pablo, Carlos Gómez, Jesús Poza

**Affiliations:** 1 Department of Clinical Neuropsychiatry, Medical University of Lublin, Lublin, Poland; 2 Department of Experimental Psychology, Institute of Psychology, The John Paul II Catholic University of Lublin, Lublin, Poland; 3 Biomedical Engineering Group, University of Valladolid, Valladolid, Spain; 4 Centro de Investigación Biomédica en Red de Bioingeniería, Biomateriales y Nanomedicina (CIBER-BBN), Instituto de Salud Carlos III, Madrid, Spain; 5 Instituto de Investigación Biosanitaria de Valladolid (IBioVALL), Valladolid, Spain; 6 Instituto de Investigacion en Matematical de la Universidad de Valladolid (IMUVA), Instituto de Investigación en Matemáticas, University of Valladolid, Valladolid, Spain

**Keywords:** early adversity, early life stress (ELS), predictive processing, statistical learning, young adult

## Abstract

**Background:**

Early adversity is associated with long-term alterations in learning and decision-making under uncertainty, yet its impact on implicit predictive processing remains insufficiently characterized. This study investigated implicit statistical learning (ISL) in relation to exposure to adverse childhood experiences, operationalized using Childhood Trauma Questionnaire (CTQ) scores, in a non-clinical sample of young adults.

**Methods:**

Fifty-four participants were classified into High-CTQ and Low-CTQ groups and completed a visual ISL task in which stimulus predictability varied across conditions. Behavioral indices included reaction time and response accuracy as measures of sensitivity to probabilistic structure.

**Results:**

Both groups exhibited an implicit learning effect, indicated by faster responses in high- versus low-predictability conditions. However, this effect was attenuated in the High-CTQ group, suggesting reduced behavioral differentiation across probabilistic contingencies. Although the group × condition interaction for reaction time was not statistically significant, a significant main effect of group emerged for accuracy, with lower performance in the High-CTQ group, particularly in the low-predictability condition. In addition, individuals with higher CTQ scores subjectively reported perceiving stimulus sequences as more random despite equivalent task engagement across groups.

**Conclusions:**

These findings suggest that early adversity is associated with altered utilization of probabilistic environmental structure during statistical learning. Rather than reflecting a generalized deficit in learning or processing speed, the observed pattern indicates moderately reduced sensitivity to weaker predictive signals and a biased subjective representation of environmental randomness in the High-CTQ group.

## 1 Introduction

Adverse childhood experiences (ACEs), including abuse, neglect, and household dysfunction, are significant developmental stressors that exert long-lasting effects on brain structure and function across the lifespan (1). ACEs are associated with increased vulnerability to psychiatric disorders (2), as well as alterations in multiple domains of neurocognitive functioning in adulthood (3, 4). Neuroimaging studies indicate that higher ACE exposure is linked to structural and functional changes in cortical and subcortical brain regions involved in executive control, memory, emotional regulation, and adaptation to environmental demands (3, 5).

Much of the research on ACE cognitive consequences has focused on memory and learning. Evidence from human and animal studies links early adversity to long-term memory impairments (6, 7), with higher ACE exposure associated with poorer episodic, verbal, and working memory (4, 7). Adversity has also been linked to altered learning under uncertainty and to differences in the use of probabilistic information (8, 9), with individuals behaving as if environmental contingencies are less stable or more random than they actually are (10). Similarly, in our previous study, adversity-exposed participants showed reduced benefit from temporal regularities in stabilizing performance (11). While these findings highlight alterations in reinforcement-based and uncertainty-related learning, they do not determine whether early adversity also affects mechanisms supporting the formation of accurate predictions, such as statistical learning.

Implicit statistical learning (SL) refers to the incidental detection of environmental regularities that support the formation of predictions about future events in the absence of explicit reinforcement (12, 13). Within the predictive processing framework, SL can be understood as a mechanism through which the brain extracts statistical structure from the environment to reduce uncertainty (14, 15). In the present study, SL is operationalized behaviorally as performance differences across probabilistic conditions, reflecting the extent to which learned regularities influence responses. To our knowledge, no previous studies have directly examined implicit statistical learning in individuals exposed to early adversity. Despite its theoretical relevance, this question therefore remains unexplored.

Neurocognitive accounts of implicit statistical learning provide a plausible link between early adversity and altered probabilistic learning. SL relies on distributed neural systems, with the hippocampus supporting the initial extraction of statistical regularities and the prefrontal cortex (PFC) contributing to the subsequent use of learned predictions (16). More generally, statistical learning is thought to depend on automatic associative mechanisms alongside an attention-dependent system involving the PFC and related working memory networks (17). Notably, these regions overlap with neural systems consistently affected by early adversity. Human and animal studies show that early-life stress is associated with hippocampal dysfunction, including altered neuroplasticity and hippocampal-dependent learning (18, 19), as well as structural changes in the hippocampus and medial PFC (20, 21). This overlap suggests that ACE-related neurodevelopmental alterations may influence the acquisition and use of environmental regularities during SL.

The present study aimed to examine whether early adversity is associated with differences in the utilization of probabilistic information during statistical learning in a non-clinical sample of young adults. Given that ISL is a fundamental and early-developing mechanism that is typically preserved across a range of clinical conditions (22, 23), we expected that early adversity would not lead to generalized impairment, but rather to subtle differences in how probabilistic information is used during ISL task performance. Importantly, given the preliminary and exploratory nature of the study, potential group differences were expected to emerge not only as condition-dependent effects reflecting differential use of probabilistic information, but also as more general differences in task performance, such as overall response speed, accuracy, or response stability. To minimize the contribution of additional cognitive demands unrelated to basic statistical learning, we employed a simple visual SL paradigm previously validated across age groups (24–26). Compared with paradigms using perceptually complex stimuli; e.g., unfamiliar visual symbols (27), longer dependency structures (e.g., the triplets; 28), or artificial language learning (29), which may additionally recruit processes such as complex visual analysis, working memory, and linguistic learning mechanisms, this task relies on the extraction of simple probabilistic regularities between sequential stimuli. Thus, it provides a more direct measure of sensitivity to environmental regularities while reducing the influence of domain-specific higher-order cognitive processes.

## 2 Methods

### 2.1 Participants

Participant recruitment involved an initial pre-screening phase. Study invitations were distributed at three universities in Lublin, Poland. Individuals interested in participating (n = 324) completed a computer-based questionnaire assessing inclusion and exclusion criteria. Inclusion criteria were: age 19–25 years, Polish native language, and student status. Exclusion criteria included receiving a disability pension; history of psychiatric treatment or psychoactive substance abuse; and neurological conditions, including head injuries, loss of consciousness, and subjective cognitive decline. Additional exclusions were neurodevelopmental disorders (e.g., dyslexia) and a history of significant academic difficulties (e.g., grade retention). Participants were also asked about any life- or health-threatening events within the previous month; events such as physical assault, traffic accidents, or sexual abuse were likewise exclusionary.

Individuals exposed to early adversity often exhibit greater negative emotionality (30) and more frequent negative thinking (31). Since negative affect and intrusive thoughts may consume cognitive resources required for efficient predictive processing (32), these variables were assessed using the Beck Depression Inventory-II (BDI-II; 33) and the White Bear Suppression Inventory (WBSI; 34), respectively. Both instruments have Polish adaptations demonstrating satisfactory reliability and validity (35, 36). The Polish WBSI adaptation shows a two-factor structure, distinguishing intrusive thought frequency from suppression attempts (36). According to Polish BDI-II norms (35), participants scoring > 20 (moderate or severe depressive symptoms) were excluded.

Eligible participants then completed the Childhood Trauma Questionnaire (CTQ-28; Polish adaptation by 37), comprising five subscales: emotional, physical, and sexual abuse, and emotional and physical neglect. The summed subscale score indexes overall early adversity exposure (38). Based on the original CTQ cut-off scores, a total score of 51 or higher, indicating moderate to severe trauma exposure, was used to classify participants into the high-CTQ group (38, 39). In contrast, a total CTQ score of 36 or below indicated low to no ACE exposure; therefore, individuals within this range were assigned to the low-CTQ group, which was demographically matched to the high-CTQ group. The present sample does not overlap with that of our previous study (11).

All participants provided written informed consent. The study was conducted in accordance with the Declaration of Helsinki and approved by the local ethics committee (KE-0254/202/09/2023).

### 2.2 Statistical learning task

**Figure 1:**
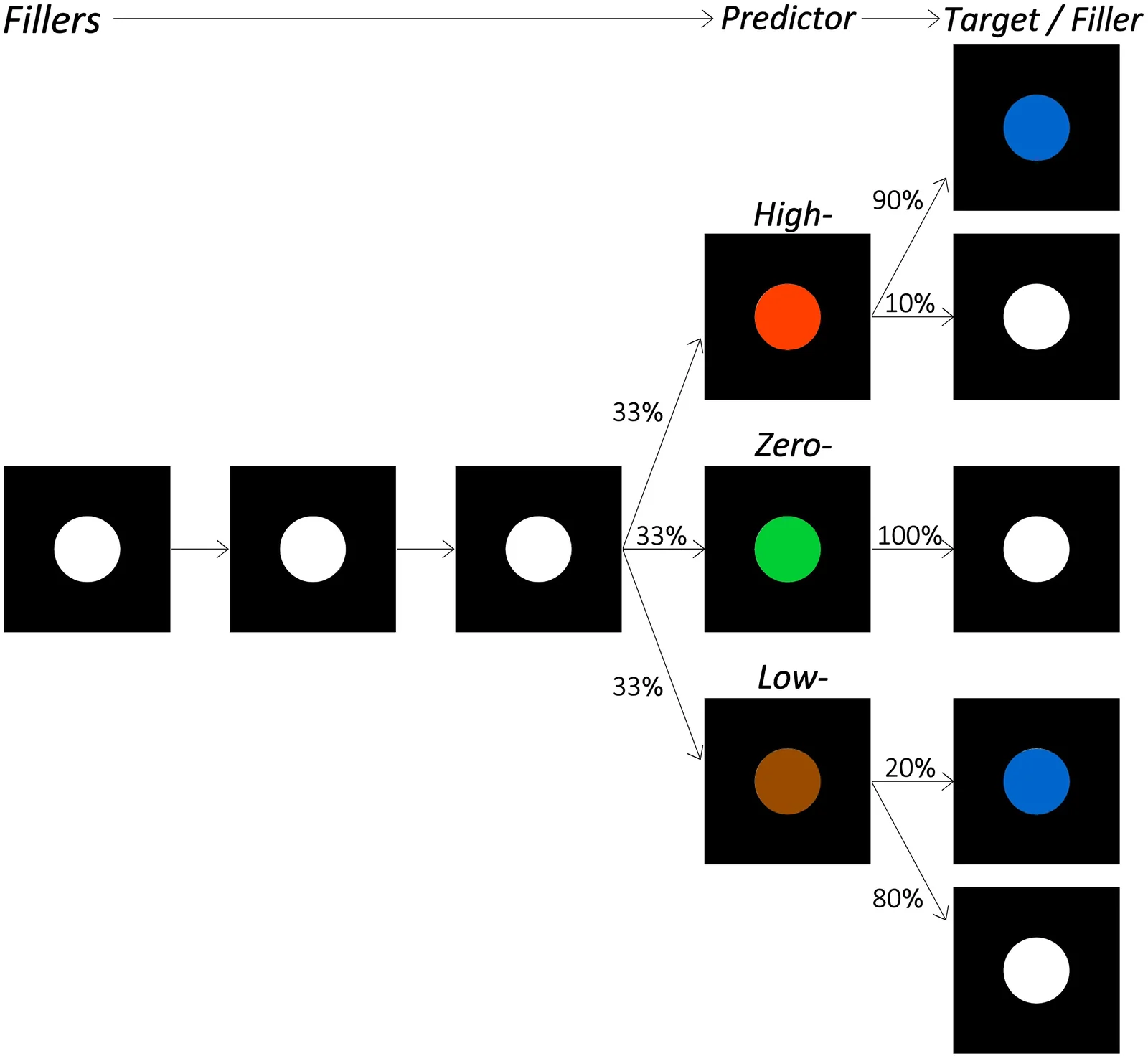
Implicit statistical learning task with an example sequence of stimuli with high, low, and zero predictability conditions. Based on Jost et al. (24).

We replicated exactly the task previously used by Jost et al. (24) and Singh et al. (26). Participants viewed a continuous sequence of colored circles presented one at a time in the center of a computer monitor against a dark background. Each stimulus was displayed for 500ms, followed by a 500ms interval with an empty black screen. Subjects were instructed to press a keyboard button whenever the target color appeared. Each trial began with one to five standard (filler) stimuli, followed by one of three predictor stimuli and the possible appearance of the target. The predictors differed in the probability of signaling the target: the high-probability predictor was followed by the target in 90% of trials, the low-probability predictor in 20% of trials, whereas the zero-probability predictor was never followed by the target, hence no target responses were noted for these trials (**Figure 1**). This structure enabled manipulation of target predictability while preserving the continuous nature of the task. Subjects were not explicitly informed about the statistical relationships between the stimuli, allowing implicit learning of predictor–target contingencies (24).

The task consisted of five blocks of 30 trials each (10 trials per predictor condition; 150 trials in total), presented in random order. Trial transitions were seamless, preventing participants from identifying clear trial boundaries. The assignment of colors (red, blue, green, brown, and white) to the predictors, standard stimulus, and target was randomized separately for each participant. Stimuli were displayed on a 15.6-inch LCD monitor (resolution 1920 × 1080 pixels, refresh rate 60 Hz) at the center of the screen (using PsychoPy v2024.2.4). The experiment took place in a quiet, well-lit room to minimize external distractions.

In this task, the primary indicator of SL effect is a significantly shorter reaction time for the high-predictability condition compared with the low-predictability condition, reflecting the extent to which participants benefit from learned contingencies (24). To complement this primary indicator, we also considered response accuracy across the two levels of predictability. Additionally, to further disentangle SL-specific effects from other cognitive influences, we analyzed response time variability as an index of potential performance noise arising from attentional lapses or reduced cognitive control.

After completing the task, participants were presented with two questions assessing subjective task experience: (1) the degree of task-related focus during performance, and (2) whether the sequence of target stimuli was perceived as random or rule-governed. Each question was displayed separately on a computer monitor, and responses were provided using a visual analog scale ranging from 1 to 100.

### 2.3 Statistical analysis

Analyses were performed using parametric and non-parametric tests as appropriate. Group differences in demographic variables, questionnaire data (CTQ, BDI-II, WBSI), and subjective task ratings were assessed using independent-samples t-tests or chi-square (χ²) tests for categorical variables. Reaction time (RT) data were pre-processed prior to analysis; RTs shorter than 50 ms were considered premature responses and excluded. RT-based analyses included only correct responses.

RT reliability in the SL task was evaluated using Cronbach’s *α* based on trial-level data separately for each group. Mean RTs were calculated for each participant separately for each condition prior to statistical analysis. Task performance was analyzed using mixed-model repeated-measures ANOVA with predictor condition (90% vs. 20% predictability) as a within-subject factor and group (High-CTQ vs. Low-CTQ) as a between-subject factor. Dependent variables included mean RT, RT variability (individual standard deviation), and response accuracy. Accuracy was defined as the proportion of correct responses across all target-present trials (0–1 scale), with 1 indicating perfect performance. This measure reflected overall performance, with both omission and commission errors reducing the final score. Effect sizes were expressed as partial eta squared (*η^2p^*). Significant effects were followed by pairwise comparisons.

Additionally, since the experimental task consisted of 5 blocks, we conducted a three-way mixed repeated-measures ANOVA on reaction time with predictability (90% vs. 20% predictability; within-subject factor), Block (1 to 5; within-subject factor), and Group (High-CTQ vs. Low-CTQ; between-subject factor).

Associations between questionnaire measures (BDI-II, WBSI) and task outcomes (mean RT, RT iSD, accuracy) were tested using Spearman correlations conducted separately within each group (n = 27). The false discovery rate (FDR) was controlled using the Benjamini–Hochberg procedure (40). Analyses were conducted using STATISTICA 13.3 software.

## 3 Results

### 3.1 Demographic characteristics and participants’ self-report data

Each final sample consisted of 27 participants, with no significant differences between them in terms of age, sex, or education (**Table 1**). The high-CTQ group scored significantly higher on the CTQ, both in the total score and across individual clinical subscales. The most pronounced differences were observed in the Emotional Abuse and Emotional Neglect subscales. None of the respondents selected the response “Very often true” on the Minimization/Denial scale item, indicating an absence of bias when completing the CTQ. BDI-II scores revealed that the high-CTQ group exhibited significantly elevated levels of depressive symptoms compared to the low-CTQ group. Although the WBSI scores were also higher in the high-CTQ group, this difference did not reach statistical significance.

**Table 1:** Demographic and self-reports of the studied groups.

Demographic data	Low-CTQ *n* = 27		High-CTQ *n* = 27		t/χ^2^	p
	M	SD	M	SD		
Age (years)	20.48	1.80	21.19	2.62	-1.152	0.254
Sex (% females)	74.10		77.80		0.440	0.507
Education (in years)	13.58	1.33	14.19	1.58	-1.010	0.344
Questionnaire data						
CTQ^1^ emotional abuse	7.22	2.27	16.69	4.61	-9.521	<0.0001
CTQ physical abuse	5.29	0.72	7.19	2.26	-4.140	0.0001
CTQ sexual abuse	5.37	1.11	7.30	3.66	-2.625	0.011
CTQ emotional neglect	8.11	3.20	15.84	3.98	-7.801	<0.0001
CTQ physical neglect	6.33	2.11	9.50	3.00	-4.447	<0.0001
CTQ total score	32.33	6.17	56.57	9.44	-11.101	<0.0001
BDI-II^2^	8.25	5.69	16.46	8.24	-4.260	0.0001
WBSI^3^ intrusive thoughts	23.14	5.58	25.84	5.83	-1.719	0.091
WBSI thoughts suppression	28.96	5.86	31.53	4.45	-1.794	0.078
WBSI total score	52.11	10.63	57.38	8.69	-1.971	0.054
Task-related subjective ratings						
Concentration^4^	71.24	18.97	72.01	21.55	-0.133	0.894
Randomness vs. regularity^5^	66.28	27.43	50.17	58.89	2.177	0.034

1 Childhood Trauma Questionnaire.

2 Beck Depression Inventory-II.

3 White Bear Suppression Inventory. The Polish WBSI version has a two-factor structure.

4 The higher the score, the better the concentration.

5 The higher the score, the stronger the belief in the target stimulus onset regularity.

Regarding subjective ratings collected after the main experimental task, participants in both groups reported similar levels of on-task concentration. However, the High-CTQ group was subjectively more convinced that the appearance of target stimuli was random and did not follow a specific rule (*p* = 0.034).

### 3.2 An analysis of the implicit statistical learning task outcomes

Task reliability analysis based on reaction time (RT) trials indicated good internal consistency in both groups, with Cronbach’s *α* values of 0.84 (High-CTQ) and 0.85 (Low-CTQ), respectively.

**Figure 2:**
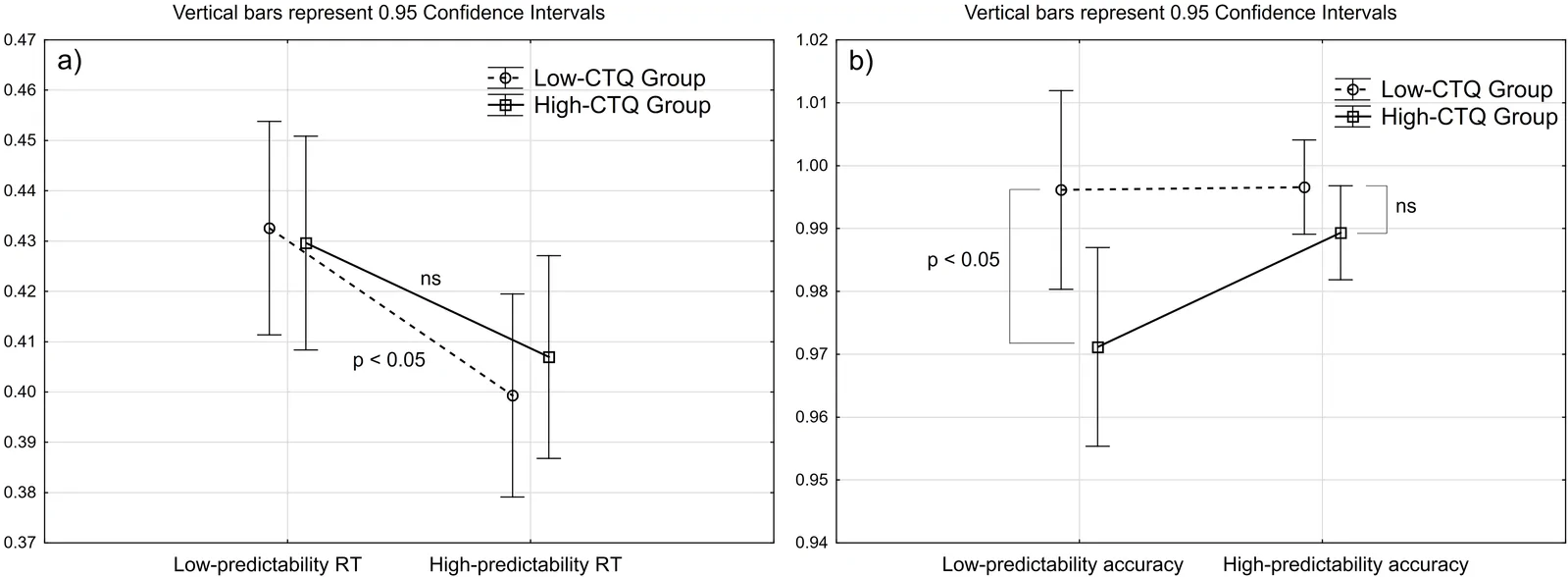
Exploratory within- and between-group comparisons. **(A)**. Within-group differences regarding mean reaction time; statistically significant difference within the Low-CTQ group, and non-significant difference in the High-CTQ group. **(B)**. Between-group differences regarding response accuracy; statistically significant group difference for the low-predictability condition, and non-significant group differences for the high-predictability condition.

As shown in **Table 2**, there was a significant main effect of condition on mean RT, with slower responses in the low-predictability condition compared to the high-predictability condition (*p* < 0.001). Exploratory *post hoc* comparisons indicated that this effect was significant in the Low-CTQ group but not in the High-CTQ group (**Figure 2A**). There was no main effect of group (*p* = 0.850), and the group × condition interaction did not reach statistical significance (*p* = 0.496). RTs for identical conditions were comparable between groups.

**Table 2:** Implicit statistical learning task analysis in the two studied groups.

Dependent variables	Groups	High-predict.		Low-predict.		Within-group effect			*Post hoc*	Between-group effect			*Post hoc*	Interaction effect		
		M	SD	M	SD	*F*	*p*	*η^2^_p_*		*F*	*p*	*η^2^_p_*		*F*	*p*	*η^2^_p_*
RT mean	Low-CTQ	0.432	0.054	0.399	0.057	12.992	<0.001	0.20	90<20 only in low-CTQ	0.036	0.850	0.01	–	0.470	0.496	0.01
	High-CTQ	0.429	0.053	0.406	0.044											
RT iSD	Low-CTQ High-CTQ	0.064	0.029	0.067	0.024	0.021	0.884	0.01	–	2.481	0.121	0.040	–	0.388	0.536	0.01
		0.079	0.033	0.077	0.043											
Accuracy	Low-CTQ	0.996	0.019	0.996	0.010	2.630	0.110	0.05	–	6.050	0.010	0.11	Low-CTQ>High-CTQ in 20% condition	2.400	0.127	0.04
	High-CTQ	0.971	0.053	0.989	0.024											

High-predict, high predictability condition; Low-predict, low predictability condition.

RT variability (individual standard deviation) did not show any significant effects.

Additionally, the block dynamic analysis was conducted. The analysis revealed a significant main effect of Predictability (*p* < 0.001; *η^2p^* = 0.24). Reaction time was longer for low predictability, *M* = 0.431; *SD* = 0.01, than for high predictability, *M* = 0.399; *SD* = 0.01. However, the main effect of Block, the Block vs Group interaction, and the Predictability vs Block interaction were non-significant (all *ps* > 0.05).

Regarding response accuracy, there was a significant main effect of group (*p* = 0.010), with higher overall accuracy in the Low-CTQ group. Exploratory *post hoc* comparisons suggested that this effect was primarily driven by lower accuracy in the low-predictability condition in the High-CTQ group (**Figure 2B**). No significant main effect of condition (*p* = 0.110) or group × condition interaction (*p* = 0.127) was observed.

### 3.3 Associations between questionnaire data and task-related variables in the studied groups

Spearman correlation analysis, conducted in separate study groups, included questionnaire variables, i.e., data from the BDI-II and WBSI, and variables related to the main experimental task, i.e., reaction times, response accuracy, and subjective ratings on concentration and target stimulus randomness.

In the High-CTQ group, no correlations exceeded the multiple-testing-corrected level of statistical significance. In the Low-CTQ group, a significant negative correlation was observed between the WBSI thought suppression factor and mean RT in the 90% condition; *r* = -0.54, *p* = 0.006.

## 4 Discussion

This study examined implicit statistical learning in non-clinical young adults with and without exposure to early adversity. We explored whether early adversity was associated with differences in the utilization of probabilistic information during implicit statistical learning. Given the exploratory nature of the study, we expected that potential group differences could emerge either as condition-dependent differences in sensitivity to statistical regularities or as more general differences in task performance. Both groups demonstrated overall sensitivity to task contingencies, reflected in faster reaction times in the high-predictability condition compared to the low-predictability condition. This effect reached the level of statistical significance in the Low-CTQ group, but not in individuals with higher exposure to early adversity. However, no significant main effect of group or group × condition interaction was observed for reaction time. Thus, the exploratory within-group comparisons should be interpreted as descriptive indications of performance patterns within each group rather than as evidence of between-group differences in statistical learning effects.

There were no overall group differences in mean reaction time or reaction time variability, suggesting comparable processing speed and response stability across groups. This indicates that the observed effects are unlikely to reflect generalized cognitive slowing or increased performance variability. Although prior studies have reported slowed processing associated with early adversity in other cognitive domains (41), the present findings suggest that such effects may not extend to this type of implicit predictive task.

A significant group effect emerged for accuracy, with the High-CTQ group showing reduced performance selectively in the low-predictability condition. Given the asymmetric structure of the task (high-predictability vs. low-predictability), these findings raise the possibility that individuals with higher adversity exposure may differ in the utilization of weaker probabilistic information. One possibility is that higher adversity exposure may be associated with subtle differences in the utilization of probabilistic information, particularly when predictive cues are less reliable. However, this interpretation remains tentative and requires confirmation in studies specifically designed to test differences in sensitivity to varying levels of contingency. Alternatively, early adversity may be associated with reduced flexibility in adjusting reliance on environmental contingencies, favoring dominant high-probability patterns even when they are less informative. This latter interpretation is broadly consistent with previous reports of reduced cognitive flexibility following early adversity (42, 43).

Subjective ratings further indicated group differences in perceived environmental structure. Participants in the High-CTQ group reported a greater tendency to perceive stimulus sequences as random, despite comparable levels of task engagement across groups. This dissociation between objective task structure and subjective experience raises the possibility that exposure to early adversity is associated not only with subtle differences in behavioral adaptation to probabilistic regularities but also with differences in how environmental structure is consciously perceived.

Regarding questionnaire measures, the High-CTQ group showed elevated depressive symptoms (BDI-II), replicating expected group differences in affective burden (44). However, depressive symptoms were not associated with task performance in either group, suggesting they did not meaningfully account for behavioral effects. In the Low-CTQ group, higher WBSI scores correlated with faster reaction times in the high-predictability condition. Given that WBSI reflects intrusive cognition and reduced thought control (36, 45), this finding may indicate that more off-task thought control is linked to faster responding under strongly predictive conditions. However, given its correlational nature and absence in the High-CTQ group, this result remains preliminary.

Importantly, the statistically significant effects identified in the present study should be interpreted in the context of an overall pattern of largely preserved behavioral performance in the High-CTQ group, as reflected by both reaction time and accuracy measures. Furthermore, the current design does not allow conclusions as to whether the statistical learning characteristics observed in this group have meaningful consequences for predictive functioning in everyday settings.

Several limitations should be considered. Although the study addresses a relatively underexplored question in a non-clinical sample, the relatively small sample size represents an important limitation, particularly for testing interaction effects, owing to limited statistical power. While the strict exclusion criteria and recruitment strategy were adopted to maximize sample homogeneity and internal validity, they do not eliminate limitations associated with the sample size. Future research with larger samples should examine whether the present findings, particularly the interaction effects, can be replicated.

Group classification relied on retrospective self-report rather than objective measures of adversity, a common but imperfect approach in ACE research (46). Additionally, it should be acknowledged that the study’s sample was limited to college students within a relatively narrow age range (19–25 years). While this demographic allowed for a highly homogeneous group, it restricts the direct generalizability of the findings to broader populations. Future research should aim to replicate these findings in other populations, including wider age ranges and individuals from different educational and socio-demographic backgrounds.

The sample also included a higher proportion of women, which may limit generalizability given evidence for sex differences in the neurocognitive effects of early adversity (47, 48). Although the task provides behavioral indices of implicit statistical learning, it does not allow direct inference about underlying computational mechanisms. Further research should also disentangle different types of early adversity, particularly threat versus deprivation (49), to better specify developmental pathways relevant to predictive processing. The relatively pronounced contrast between the predictive conditions employed in the present task (high-predictability vs. low-predictability) may have facilitated the successful extraction of environmental regularities in both groups, thereby limiting sensitivity to subtle between-group differences.

The characteristics of the statistical learning paradigm may influence which aspects of predictive learning are captured. The task used in our study involved emotionally neutral visual stimuli and relatively simple probabilistic relationships, allowing us to focus on basic sensitivity to environmental regularities. However, early adversity may be particularly relevant for learning processes involving emotionally or motivationally significant information. Given that adverse environments can shape attention toward threat- or safety-related cues (50), paradigms incorporating affectively salient stimuli may reveal differences in how environmental regularities are detected, prioritized, and used for prediction. Finally, tasks involving weaker or more ambiguous probabilistic contingencies (e.g., 60% vs. 40%) may provide a more sensitive measure for an adversity group selected from the general population, without major cognitive deficits, and show processes of accumulating evidence, updating predictions, and responding to uncertainty. Thus, future research comparing different types of statistical learning paradigms may help clarify whether early adversity is associated with broad alterations in predictive learning or with context-dependent differences that emerge under specific environmental demands.

In summary, the present findings provide preliminary evidence that exposure to early adversity may be associated with differences in task performance and subjective perception during an implicit statistical learning paradigm, while overall behavioral sensitivity to probabilistic structure appears largely preserved. Given the absence of robust group × condition interactions, conclusions regarding adversity-related differences in the mechanisms of statistical learning remain tentative and require replication.

## Data Availability

The raw data supporting the conclusions of this article will be made available by the authors, without undue reservation.

## References

[1] Heany SJ Groenewold NA Uhlmann A Dalvie S Stein DJ Brooks SJ . The neural correlates of childhood trauma questionnaire scores in adults: a meta- analysis and review of functional magnetic resonance imaging studies. Dev Psychopathol. (2018) 30:1475–85. doi: 10.1017/S0954579417001717 29224580

[2] Grummitt L Baldwin JR Lafoa'i J Keyes KM Barrett EL . Burden of mental disorders and suicide attributable to childhood maltreatment. JAMA Psychiatry. (2024) 81:782–8. doi: 10.1001/jamapsychiatry.2024.0804 38717764 PMC11079790

[3] Bick J Nelson CA . Early adverse experiences and the developing brain. Neuropsychopharmacology. (2016) 41:177–96. doi: 10.1038/npp.2015.252 26334107 PMC4677140

[4] Hawkins MAW Layman HM Ganson KT Tabler J Ciciolla L Tsotsoros CE . Adverse childhood events and cognitive function among young adults: Prospective results from the national longitudinal study of adolescent to adult health. Child Abuse Negl. (2021) 115:105008. doi: 10.1016/j.chiabu.2021.105008 33706023 PMC11610377

[5] Klimesch A Ascone L Thomalla G Cheng B Petersen M Schäfer I . Echoes of childhood trauma: the relationship between adverse childhood experiences, brain structure, and mental health in aging adults. Transl Psychiatry. (2026) 16:52. doi: 10.1038/s41398-026-03811-2 41633982 PMC12873319

[6] Molet J Maras PM Kinney-Lang E Harris NG Rashid F Ivy AS . MRI uncovers disrupted hippocampal microstructure that underlies memory impairments after early-life adversity. Hippocampus. (2016) 26:1618–32. doi: 10.1002/hipo.22661 27657911 PMC5452614

[7] Johnson KE Brown MJ . The long-term impact of adverse childhood experiences on episodic memory in mid- to late-life among US older adults. Aging Ment Health. (2026) 27:1–11. doi: 10.1080/13607863.2026.2621787 41593018 PMC12990772

[8] Weller JA Fisher PA . Decision-making deficits among maltreated children. Child Maltreat. (2013) 18:184–94. doi: 10.1177/1077559512467846 23220788 PMC3737421

[9] Armbruster-Genç DJN Neil L Valton V Phillips H Rankin G Sharp M . Childhood maltreatment is associated with lower exploration and disrupted prefrontal activity and connectivity during reward learning in volatile environments. J Child Psychol Psychiatry. (2025) 66:846–56. doi: 10.1111/jcpp.14095 39665511 PMC12062857

[10] Hanson JL van den Bos W Roeber BJ Rudolph KD Davidson RJ Pollak SD . Early adversity and learning: implications for typical and atypical behavioral development. J Child Psychol Psychiatry. (2017) 58:770–8. doi: 10.1111/jcpp.12694 28158896 PMC5474156

[11] Krukow P Kopiś-Posiej N Pablo VG Rodríguez-González V Gómez C Poza J . Altered task-related brain network dynamics and performance consistency in a non-clinical group burdened with childhood trauma. Prog Neuro-Psychopharmacol Biol Psychiatry. (2025) 143:111571. doi: 10.1016/j.pnpbp.2025.111571 41349875

[12] Saffran J . Statistical learning. In: Frank MC Majid A , editors. Open Encyclopedia of Cognitive Science. Cambridge: MIT Press (2024). doi: 10.21428/e2759450.7a74eed8

[13] Conway CM Christiansen MH . Statistical learning within and between modalities: Pitting abstract against stimulus-specific representations. Psychol Sci. (2006) 17:905–12. doi: 10.1111/j.1467-9280.2006.01801.x 17100792

[14] Friston K . The free-energy principle: a unified brain theory? Nat Rev Neurosci. (2010) 11:127–38. doi: 10.1038/nrn2787 20068583

[15] Clark A . Whatever next? Predictive brains, situated agents, and the future of cognitive science. Behav Brain Sci. (2013) 36:181–204. doi: 10.1017/S0140525X12000477 23663408

[16] Spaak E de Lange FP . Hippocampal and prefrontal theta-band mechanisms underpin implicit spatial context learning. J Neurosci. (2020) 40:191–202. doi: 10.1523/JNEUROSCI.1660-19.2019 31699887 PMC6939492

[17] Conway CM . How does the brain learn environmental structure? Ten core principles for understanding the neurocognitive mechanisms of statistical learning. Neurosci Biobehav Rev. (2020) 112:279–99. doi: 10.1016/j.neubiorev.2020.01.032 32018038 PMC7211144

[18] Hoeijmakers L Lucassen PJ Korosi A . The interplay of early-life stress, nutrition, and immune activation programs adult hippocampal structure and function. Front Mol Neurosci. (2015) 7:103. doi: 10.3389/fnmol.2014.00103 25620909 PMC4288131

[19] Pillai AG Arp M Velzing E Lesuis SL Schmidt MV Holsboer F . Early life stress determines the effects of glucocorticoids and stress on hippocampal function: Electrophysiological and behavioral evidence respectively. Neuropharmacology. (2018) 133:307–18. doi: 10.1016/j.neuropharm.2018.02.001 29412144

[20] Gorka AX Hanson JL Radtke SR Hariri AR . Reduced hippocampal and medial prefrontal gray matter mediate the association between reported childhood maltreatment and trait anxiety in adulthood and predict sensitivity to future life stress. Biol Mood Anxiety Disord. (2014) 4:12. doi: 10.1186/2045-5380-4-12 25408863 PMC4236295

[21] Smith KE Pollak SD . Early life stress and development: potential mechanisms for adverse outcomes. J Neurodev Disord. (2020) 12:34. doi: 10.1186/s11689-020-09337-y 33327939 PMC7745388

[22] Parks KMA Stevenson RA . Auditory and visual statistical learning are not related to ADHD symptomatology: Evidence from a research domain criteria (RDoC) approach. Front Psychol. (2018) 9:2502. doi: 10.3389/fpsyg.2018.02502 30618933 PMC6308122

[23] Bell RR Thomas HR Saffran JR Eigsti IM . A systematic review of statistical learning in autism spectrum disorder. Mol Autism. (2025) 17:2. doi: 10.1186/s13229-025-00697-7 41437281 PMC12805773

[24] Jost E Conway CM Purdy JD Walk AM Hendricks MA . Exploring the neurodevelopment of visual statistical learning using event-related brain potentials. Brain Res. (2015) 1597:95–107. doi: 10.1016/j.brainres.2014.10.017 25475992 PMC4340771

[25] Daltrozzo J Emerson SN Deocampo J Singh S Freggens M Branum-Martin L . Visual statistical learning is related to natural language ability in adults: An ERP study. Brain Lang. (2017) 166:40–51. doi: 10.1016/j.bandl.2016.12.005 28086142 PMC5293669

[26] Singh S Walk AM Conway CM . Atypical predictive processing during visual statistical learning in children with developmental dyslexia: an event-related potential study. Ann Dyslexia. (2018) 68:165–79. doi: 10.1007/s11881-018-0161-2 29907920 PMC6390967

[27] Ren J Wang M . Contribution of statistical learning in learning to read across languages. PloS One. (2024) 19:e0298670. doi: 10.1371/journal.pone.0298670 38527080 PMC10962809

[28] Parks KMA Griffith LA Armstrong NB Stevenson RA . Statistical learning and social competency: The mediating role of language. Sci Rep. (2020) 10:3968. doi: 10.1038/s41598-020-61047-6 32132635 PMC7055309

[29] Franco A Cleeremans A Destrebecqz A . Statistical learning of two artificial languages presented successively: How conscious? Front Psychol. (2011) 2:229. doi: 10.3389/fpsyg.2011.00229 21960981 PMC3177082

[30] Gardner MJ Thomas HJ Erskine HE . The association between five forms of child maltreatment and depressive and anxiety disorders: A systematic review and meta-analysis. Child Abuse Negl. (2019) 96:104082. doi: 10.1016/j.chiabu.2019.104082 31374447

[31] Mansueto G Cavallo C Palmieri S Ruggiero GM Sassaroli S Caselli G . Adverse childhood experiences and repetitive negative thinking in adulthood: A systematic review. Clin Psychol Psychother. (2021) 28:557–68. doi: 10.1002/cpp.2590 33861493

[32] Eysenck MW Derakshan N Santos R Calvo MG . Anxiety and cognitive performance: Attentional control theory. Emotion. (2007) 7:336–53. doi: 10.1037/1528-3542.7.2.336 17516812

[33] Beck AT Steed RA Brown GK . Manual for the Beck Depression Inventory-II. San Antonio, TX: Psychological Corporation (1996).

[34] Wegner DM Zanakos S . Chronic thought suppression. J Pers. (1994) 62:616–40. doi: 10.1111/j.1467-6494.1994.tb00311.x 7861307

[35] Łojek E Stańczak J . Inwentarz Depresji Becka – Drugie Wydanie [Beck Depression Inventory – Second Edition; Bdi-Ii]. Warszawa: Pracownia Testów Psychologicznych PTP (2019).

[36] Cichoń E Szczepanowski R Niemiec T . Polish version of the White Bear Suppression Inventory (WBSI) by Wegner and Zanakos: factor analysis and reliability. Psychiatr Pol. (2020) 54:125–35. doi: 10.12740/PP/93493 32447361

[37] Murzyn A . Childhood Trauma Experience as a Predicate of Therapeutic Response in Patients with Neurotic and Personality Disorders. PhD dissertation. Kraków: Jagiellonian University (2012).

[38] Bernstein DP Stein JA Newcomb MD Walker E Pogge D Ahluvalia T . Development and validation of a brief screening version of the childhood trauma questionnaire. Child Abuse Negl. (2003) 27:169–90. doi: 10.1016/s0145-2134(02)00541-0 12615092

[39] Bernstein DP Fink L . Childhood Trauma Questionnaire. A Retrospective Self- Report Questionnaire and Manual. San Antonio: The Psychological Corporation (1998).

[40] Benjamini Y Drai D Elmer G Kafkafi N Golani I . Controlling the false discovery rate in behavior genetics research. Behav Brain Res. (2001) 125:279–84. doi: 10.1016/s0166-4328(01)00297-2 11682119

[41] Vermeent S . Adversity is associated with lower general processing speed rather than executive functioning. J Exp Psychol Gen. (2025) 154:3010–28. doi: 10.1037/xge0001812 40892598

[42] Spann MN Mayes LC Kalmar JH Guiney J Womer FY Pittman B . Childhood abuse and neglect and cognitive flexibility in adolescents. Child Neuropsychol. (2012) 18:182–9. doi: 10.1080/09297049.2011.595400 21942637 PMC3326262

[43] Kalia V Knauft K Hayatbini N . Adverse childhood experiences (ACEs) associated with reduced cognitive flexibility in both college and community samples. PloS One. (2021) 16:e0260822. doi: 10.1371/journal.pone.0260822 34855895 PMC8638954

[44] Yu Z Cao Y Shang T Li P . Depression in youths with early life adversity: a systematic review and meta-analysis. Front Psychiatry. (2024) 15:1378807. doi: 10.3389/fpsyt.2024.1378807 39328345 PMC11424519

[45] Muris P Merckelbach H Horselenberg R . Individual differences in thought suppression. The White Bear Suppression Inventory: factor structure, reliability, validity and correlates. Behav Res Ther. (1996) 34:501–13. doi: 10.1016/0005-7967(96)00005-8 8687372

[46] Rosa M Scassellati C Cattaneo A . Association of childhood trauma with cognitive domains in adult patients with mental disorders and in non-clinical populations: a systematic review. Front Psychol. (2023) 14:1156415. doi: 10.3389/fpsyg.2023.1156415 37425159 PMC10327487

[47] Garvin MM Bolton JL . Sex-specific behavioral outcomes of early-life adversity and emerging microglia-dependent mechanisms. Front Behav Neurosci. (2022) 16:1013865. doi: 10.3389/fnbeh.2022.1013865 36268470 PMC9577368

[48] Wolfova K Csajbok Z Kagstrom A Kåreholt I Cermakova P . Role of sex in the association between childhood socioeconomic position and cognitive ageing in later life. Sci Rep. (2021) 11:4647. doi: 10.1038/s41598-021-84022-1 33633200 PMC7907064

[49] McLaughlin KA Sheridan MA Lambert HK . Childhood adversity and neural development: deprivation and threat as distinct dimensions of early experience. Neurosci Biobehav Rev. (2014) 47:578–91. doi: 10.1016/j.neubiorev.2014.10.012 25454359 PMC4308474

[50] Smith KE Lillian Xu Y Pollak SD . How childhood adversity affects components of decision making. Neurosci Biobehav Rev. (2025) 169:106027. doi: 10.1016/j.neubiorev.2025.106027 39870319 PMC12358036

